# Supplementary material for: Discovery of genomic and transcriptomic pleiotropy between kidney function and soluble receptor for advanced glycation end products using correlated meta‐analyses: The Long Life Family Study
Source: Aging Cell. 2024 Jun 26;23(10):e14261. doi: 10.1111/acel.14261 (PMC11464144; doi:10.1111/acel.14261)
Supplement: Supplementary file 1 — Data S1. [file ACEL-23-e14261-s002.docx]

**Discovery of genomic and transcriptomic pleiotropy between kidney function and soluble receptor for advanced glycation end-products using correlated meta-analyses: The Long Life Family Study**

Feitosa MF et al.

**SUPPLEMENTAL MATERIAL**

**Appendix.** Additional discussion for novel associated loci with kidney-related traits or longevity (pg. 2)

**Supplemental Tables**

**Supplemental Table 1.** Characteristics of participants in the analyses (pg. 5)

**Supplemental Table 2.** Distribution of creatinine, cystatin C, and sRAGE by age groups (pg. 7)

**Supplemental Table** **3**. Genomic control (λ) of GWAS and CMA for kidney function and sRAGE (pg. 8)

**Supplemental Table 4.** Tetrachoric correlations of CMA for kidney function traits and sRAGE (pg. 8)

**Supplemental Table 5.** Novel loci from CMA GWAS for kidney function, GWAS catalog, and TCGA (xlxs)

**Supplemental Table 6.** Reported loci for kidney function and replicated by CMA GWAS (xlsx)

**Supplemental Table 7.** NHGRI-EBI GWAS catalog for kidney function loci replicated by CMA GWAS (xlsx)

**Supplemental Table 8.** HaploReg regulatory features for locus variants identified by CMA GWAS (xlsx)

**Supplemental Table 9.** GTEx and Human Kidney eQTL Atlas (xlsx)

**Supplemental Table 10.** CMA TWAS, Human Kidney eQTL Atlas, and TCGA (xlsx)

**Supplemental Table 11.** CMA TWAS, GeneHancer-GeneCards, and GWAS Catalog (xlsx)

**Supplemental Table 12.** EPRI-ICGC, GWAS catalog, and CMA GWAS (xlsx)

**Supplemental Table 13.** Summary of literature for genes identified by CMA TWAS (xlsx)

**Supplemental Table 14.** Comparison of CKD between LLFS and random US populations (pg. 9)

**Supplemental Figures**

**Supplemental Figure 1.** Distribution of creatinine, cystatin C and sRAGE by age group boxplots (pg. 10)

**Supplemental Figure 2.** GWAS quantile-quantile plots of observed vs. expected -log_10_ (*p-*value) (pg. 11)

**Supplemental Figure 3.** Manhattan plots of GWAS for kidney function traits and sRAGE (pg. 12)

**Supplemental Figure 4.** Manhattan plots of CMA GWAS for kidney function traits and sRAGE (pg. 13)

**Supplemental Figure 5.** Locuszoom plots of novel CMA GWAS (pg. 14)

**Supplemental Figure 6.** TWAS quantile-quantile plots of observed vs expected -log_10_ (*p*-value) (pg. 21)

**Supplemental Figure 7.** Manhattan plots of TWAS for kidney function traits and sRAGE (pg. 22)

**Appendix.** Additional discussion for novel associated loci with kidney-related traits or longevity

Correlated meta-analyses (CMA) on genome-wide association study (GWAS) *p*-values enable the identification of 42 novel pleiotropic loci for kidney function and sRAGE. Eleven of the 42 novel loci, which were previously associated with kidney-related traits (1p32.3, 1p22.2, 4p16.2, 4q34.3, 7q35-q36.1, 9q22.33, 17q25.3, and 18p11.23, Supplemental Table 4) or longevity (12q24.33, 17q25.3, and 18q21.1-q21.2) in the GWAS catalog but not with eGFRcr and eGFRcys, were involved in kidney diseases, kidney anomalies, and aging- and kidney-related signaling pathways.

Associations on chromosome 1p32.3 were reported between *CDKN2C* and *FAF1* with renal cell carcinoma, blood urea nitrogen levels, and serum uric acid levels. *CDKN2C* and *FAF1* predicted the prognosis of KIRC and KIRP, according to the TCGA database. *FAF1* was also involved in mediating apoptosis, nuclear factor (NF)-κB, Wnt/β-catenin and transforming growth factor (TGF)-β [signaling pathways](https://www.sciencedirect.com/topics/biochemistry-genetics-and-molecular-biology/signal-transduction), [mineralocorticoid receptor](https://www.sciencedirect.com/topics/biochemistry-genetics-and-molecular-biology/mineralocorticoid-receptor)-mediated [transactivation](https://www.sciencedirect.com/topics/biochemistry-genetics-and-molecular-biology/transactivation), and ubiquitin-dependent processes ([C. H. Wang et al., 2019](#_ENREF_12)).

At 1p22.2 locus, *LRRC8C* was associated with serum uric acid levels. The LRRC8C protein is a critical component of T cells' volume-regulated anion channel (VRAC). The VRAC/LRRC8C suppresses T cell function, controlling T cell-mediated immune response by regulating cyclic dinucleotide transport and STING (stimulator of IFN genes)–p53 signaling ([Concepcion et al., 2022](#_ENREF_3)). VRAC/LRRC8 channels are also crucial for the function and integrity of proximal tubules. In mouse models, *lrrc8d* was prominently expressed in renal vascular endothelial cells, and its protein was colocalized with the endothelial marker ICAM1 in the outer medulla and glomerulus ([Lopez-Cayuqueo et al., 2022](#_ENREF_9)). *LRRC8D* also predicted KIRC and KIRP in the TCGA database.

At 17q25.3 locus, associations were previously identified between *SOCS3/LINC01993* variants with creatine kinase levels and *TMC8* with serum creatinine and uric acid levels. *TMC8* is downstream regulated by microRNA‑144‑5p/oncogenic syndecan‑3 axes associated with a poor prognosis of renal clear cell carcinoma (RCC) ([Yamada et al., 2018](#_ENREF_14)). The other two-locus genes at 17q25.3, *TK1* and *BIRC5*, are biomarkers for predicting RCC prognosis ([Bratu et al., 2021](#_ENREF_2); [J. Wang et al., 2022](#_ENREF_13)). *TMC8*, *TK1*, and *BIRC5* also predicted poor RCC prognosis according to the TCGA database.

At the 4q34.3 locus, an intergenic variant was associated with the uric acid elevation in response to the thiazide-like diuretic in hypertension. The CMA GWAS for eGFRcr and eGFRcys identified 16 SNPs within a gene desert region on chromosome 4q34.3 (Figure 1, Supplemental Table 4). Approximately 1,240 Kb downstream of the lead SNP rs72715959 (MAF=0.0729) resides the closest gene, the lncRNA-240 (*LINC00290*), and at ~596 Kb downstream of rs777345385 (MAF=0.0029) locates the lncRNA-1098 (*LINC01098*). The terminal deletion on the 4q chromosome leads to a recognizable syndrome, including 4q34.3 deletion, with evidence of kidney anomaly ([Rossi et al., 2009](#_ENREF_11)) and autoimmune nephropathy ([Al-Owain et al., 2010](#_ENREF_1)).

At 9q22.33, an intergenic variant showed a suggestive association with eGFR in CKD patients. *CDC14B* (9q22.33) was strongly expressed in the apical proximal tubules in the nonneoplastic tissues, but its expression was completely absent in RCC cases ([Kim et al., 2014](#_ENREF_8)). The protein encoded by *CDC14B* antagonizes *CDK1*-mediated activating mitotic phosphorylation of the deubiquitinase *USP9X*, which targeted the Wilms’ tumor protein-1 (WT1) ([Dietachmayr et al., 2020](#_ENREF_5)). The mutation of transcription factor *WT1* was reported to contribute to ∼15% of aggressive pediatric kidney cancer ([Hastie, 2017](#_ENREF_6)).

In addition, associations were described between *STK32B* (4p16.2) with urate levels, *CNTNAP2* (7q35-q36.1) with diabetic kidney disease, and *PTPRM* (18p11.23) with dialysis survival ([Concepcion et al., 2022](#_ENREF_3)). The *STK32B*, *CNTNAP2*, and *PTPRM* genes predicted KIRC, and *PTPRM* also predicted KIRP in the TCGA database; however, the mechanisms involving *STK32B*, *CNTNAP2*, and *PTPRM* with kidney disease are unknown.

Moreover, three loci (12q24.33, 17q25.3, and 18q21.1-q21.2) identified from CMA GWAS for kidney function were also associated with longevity in the GWAS catalog (Supplemental Table 4). At 12q24.33 (Figure 1), the *RIMBP2* gene encodes a presynaptic protein involved in synaptic transmitter release at central synapses and also predicted KIRP and KIRC in the TCGA database. Mutations in TCF4 were linked to dysregulation of *RIMBP2,* provoking several neurodevelopmental diseases and disrupting synaptic function in patient-derived cortical neurons, such as Pitt-Hopkins syndrome ([Davis et al., 2023](#_ENREF_4)). *TCF4* is a vital transcriptional regulator of human synaptic development, function, and plasticity ([Davis et al., 2023](#_ENREF_4)). It can also increase the severity of renal injury and contribute to the apoptosis of NRK-52E renal proximal tubular epithelial cells ([Hu et al., 2021](#_ENREF_7)). However, whether *RIMBP2* participates with *TCF4* in kidney diseases is unknown.

*SKA1* (18q21.1-q21.2, Figure 1) belongs to a microtubule-binding subcomplex of the outer kinetochore, which is essential for proper chromosome segregation and is involved in the growth and proliferation of numerous cancer types, including the pathogenesis of renal cell carcinoma ([Pu et al., 2022](#_ENREF_10)). *SKA1* also predicted KIRC in the TCGA database. *LINC01987/LINC01973* (17q25.3) locus was also significantly associated with kidney-related traits and longevity. Some genes in 17q25.3, such as *BIRC5* and *TK1*, predicted kidney carcinoma in the TCGA database, but their roles remain unclear.

**References**

Al-Owain, M., Kaya, N., Al-Zaidan, H., Bin Hussain, I., Al-Manea, H., Al-Hindi, H., . . . Al-Muhsen, S. (2010). Renal failure associated with APECED and terminal 4q deletion: evidence of autoimmune nephropathy. *Clin Dev Immunol, 2010*, 586342. doi:10.1155/2010/586342

Bratu, O., Mischianu, D., Marcu, D., Spinu, D., Iorga, L., Cherciu, A., . . . Anghel, R. (2021). Renal tumor biomarkers (Review). *Exp Ther Med, 22*(5), 1297. doi:10.3892/etm.2021.10732

Concepcion, A. R., Wagner, L. E., 2nd, Zhu, J., Tao, A. Y., Yang, J., Khodadadi-Jamayran, A., . . . Feske, S. (2022). The volume-regulated anion channel LRRC8C suppresses T cell function by regulating cyclic dinucleotide transport and STING-p53 signaling. *Nat Immunol, 23*(2), 287-302. doi:10.1038/s41590-021-01105-x

Davis, B. A., Chen, H. Y., Ye, Z., Ostlund, I., Tippani, M., Das, D., . . . Maher, B. J. (2023). TCF4 mutations disrupt synaptic function through dysregulation of RIMBP2 in patient-derived cortical neurons. *bioRxiv*. doi:10.1101/2023.01.19.524788

Dietachmayr, M., Rathakrishnan, A., Karpiuk, O., von Zweydorf, F., Engleitner, T., Fernandez-Saiz, V., . . . Bassermann, F. (2020). Antagonistic activities of CDC14B and CDK1 on USP9X regulate WT1-dependent mitotic transcription and survival. *Nat Commun, 11*(1), 1268. doi:10.1038/s41467-020-15059-5

Hastie, N. D. (2017). Wilms' tumour 1 (WT1) in development, homeostasis and disease. *Development, 144*(16), 2862-2872. doi:10.1242/dev.153163

Hu, J. M., He, L. J., Wang, P. B., Yu, Y., Ye, Y. P., & Liang, L. (2021). Antagonist targeting miR‑106b‑5p attenuates acute renal injury by regulating renal function, apoptosis and autophagy via the upregulation of TCF4. *Int J Mol Med, 48*(3). doi:10.3892/ijmm.2021.5002

Kim, Y., Choi, J. W., Lee, J. H., & Kim, Y. S. (2014). Loss of CDC14B expression in clear cell renal cell carcinoma: meta-analysis of microarray data sets. *Am J Clin Pathol, 141*(4), 551-558. doi:10.1309/AJCP4PE4JPSRGBQS

Lopez-Cayuqueo, K. I., Planells-Cases, R., Pietzke, M., Oliveras, A., Kempa, S., Bachmann, S., & Jentsch, T. J. (2022). Renal Deletion of LRRC8/VRAC Channels Induces Proximal Tubulopathy. *J Am Soc Nephrol, 33*(8), 1528-1545. doi:10.1681/ASN.2021111458

Pu, Y., Han, J., Zhang, M., Liu, M., Abdusamat, G., & Liu, H. (2022). SKA1 promotes tumor metastasis via SAFB-mediated transcription repression of DUSP6 in clear cell renal cell carcinoma. *Aging (Albany NY), 14*(23), 9679-9698. doi:10.18632/aging.204418

Rossi, M. R., DiMaio, M. S., Xiang, B., Lu, K., Kaymakcalan, H., Seashore, M., . . . Li, P. (2009). Clinical and genomic characterization of distal duplications and deletions of chromosome 4q: study of two cases and review of the literature. *Am J Med Genet A, 149A*(12), 2788-2794. doi:10.1002/ajmg.a.33088

Wang, C. H., Hung, P. W., Chiang, C. W., Lombes, M., Chen, C. H., Lee, K. H., . . . Lin, D. Y. (2019). Identification of two independent SUMO-interacting motifs in Fas-associated factor 1 (FAF1): Implications for mineralocorticoid receptor (MR)-mediated transcriptional regulation. *Biochim Biophys Acta Mol Cell Res, 1866*(8), 1282-1297. doi:10.1016/j.bbamcr.2019.03.014

Wang, J., Chen, M., Dang, C., Zhang, H., Wang, X., Yin, J., . . . Zhang, Y. (2022). The Early Diagnostic and Prognostic Value of BIRC5 in Clear-Cell Renal Cell Carcinoma Based on the Cancer Genome Atlas Data. *Urol Int, 106*(4), 344-351. doi:10.1159/000517310

Yamada, Y., Arai, T., Kojima, S., Sugawara, S., Kato, M., Okato, A., . . . Seki, N. (2018). Regulation of antitumor miR-144-5p targets oncogenes: Direct regulation of syndecan-3 and its clinical significance. *Cancer Sci, 109*(9), 2919-2936. doi:10.1111/cas.13722

**Supplemental Table 1.** Characteristics of participants in the analyses

| Variables | TOTAL Percentage or Mean (SD) [range], *****median (Q1, Q3) | PROBAND  Percentage or Mean (SD) [range], *****median (Q1, Q3) | OFFSPRING Percentage or Mean (SD) [range], *****median (Q1, Q3) | SPOUSES Percentage or Mean (SD) [range], *****median (Q1, Q3) |
| --- | --- | --- | --- | --- |
| Number | 4182 | 1261 | 2094 | 827 |
| Age (years) [range] | 70.4 (15.7) [24 - 110] | 90.3 (6.4) [49-110] | 60.5 (8.2) [32-88] | 65.4 (12.0) [24-98] |
| Sex (male) | 45% | 49% | 42% | 49% |
| Body mass index (kg/m^2^) | 27.1 (4.7) [13-57] | 26.0 (4.1) [13-49] | 27.6 (5.1) [17-57] | 27.4 (4.5) [17-45] |
| Total cholesterol (mg/dL) | 199.2 (41.6) [62-379] | 186.9 (43.1) [62-347] | 205.0 (39.5) [81-379] | 203.2 (40.6) [81-379] |
| High-density lipoprotein (HDL) cholesterol (mg/dL) | 58.9 (17.2) [17-150] | 55.8 (15.7) [21-141] | 60.6 (18.0) [17-150] | 59.1 (16.7) [18-134] |
| Systolic blood pressure (mmHg) | 131.5 (22.3) [73-239] | 138.4 (25.6) [73-227] | 127.4 (19.2) [76-229] | 131.2 (21.3) [85-239] |
| Diastolic blood pressure (mmHg) | 77.1 (11.4) [42-131] | 73.4 (11.9) [42-121] | 78.8 (10.7) [47-131] | 78.5 (11.0) [45-119] |
| Hypertension | 52% | 66.9% | 42.7% | 53.3% |
| T2D | 6% | 8% | 4% | 6% |
| CHD | 9% | 18% | 4% | 8% |
| Serum sRAGE (pg/mL) | 631.21 (506.77)  [28-8721]  517.00 (371.00, 728.00) * | 820.56 (625.53)  [128-7944]  677.5 (483.5, 962.5)* | 542.65 (412.72)  [28-8721]  467 (346, 631)* | 566.88 (439.11)  [38-6770]  471 (345, 649)* |
| Serum creatinine (mg/dL) | 1.05 (0.33)  [0.33-6.81]  1.00 (0.87, 1.17) ***** | 1.20 (0.44)  [0.33-5.83]  1.12 (0.94, 1.35)* | 0.98 (0.25)  [0.33-6.81]  0.96 (0.84, 1.09)* | 1.01 (0.22)  [0.52-2.72]  1 (0.87, 1.13)* |
| eGFRcr (ml/min/1.73 m^2^) | 69.78 (18.42)  [15.00-131.02]  70.65 (58.03,82.53) ***** | 54.74 ± 16.27  [15.00-115.45]  54.19 (43.33,65.83) ***** | 77.22 ± 14.83  [15.00-131.02]  76.78 (67.20,87.71) ***** | 73.86 ± 15.92  [23.92-114.53]  74.25 (63.67,85.02) ***** |
| Serum cystatin C (mg/L) | 1.08 (0.43)  [0.4-6.18]  0.95 (0.81, 1.20)* | 1.45 (0.51)  [0.65-6.18]  1.35 (1.1, 1.66)* | 0.89 (0.26)  [0.4-5.61]  0.85 (0.76, 0.97)* | 0.97 (0.28)  [0.54-2.95]  0.9 (0.79, 1.06)* |
| eGFRcys (ml/min/1.73 m^2^) | 75.15 (27.15)  [15.00-153.83]  78.54 (53.61, 98.40)* | 47.23 (18.46)  [15.00-108.10]  44.84 (33.45, 58.71)* | 89.20 (19.35)  [15.00-153.83]  91.36 (77.11, 104.17*) | 82.13 (22.86)  [16.10-125.93]  84.92 (66.84, 100.46)* |
| CKDcr cases (N, %) | 1182 (28.26%) | 791 (62.73%) | 232 (11.08%) | 159 (19.23%) |
| CKDcys cases (N, %) | 1286 (30.75%) | 967 (76.69%) | 166 (7.93%) | 153 (18.50%) |

*Note:* Proband denominates the family members in the first generation, which includes siblings. The specifications are: mean (SD) = mean levels (standard deviation) and range (minimum and maximum values); median (first (Q1) and third (Q3) quartiles); hypertension = blood pressure (BP) above 140/90 mm Hg and/or taking anti-hypertensive medications; T2D = Type 2 diabetes; CHD = coronary heart disease; sRAGE = serum levels of soluble receptor for advanced glycation end products; eGFR = estimated glomerular filtration rate (eGFR), eGFRcr = eGFR from serum creatinine, eGFRcys = eGFR from serum cystatin C; CKDcr = chronic kidney disease (CKD) defined as an eGFRcr below 60 ml/min/1.73 m^2^; CKDcys = CKD defined as an eGFRcys below 60 ml/mi/1.73 m^2^. The mean ± SD of the difference of eGFRcys – eGFRcr: 5.37 ± 17.46 ml/min/1.73 m^2^.

**Supplemental Table 2.** Distribution of creatinine, cystatin C, and sRAGE by age groups

**Supplemental Table 3.** Genomic control (λ) of GWAS and CMA for kidney function and sRAGE

|  | eGFRcr | eGFRcys | sRAGE |
| --- | --- | --- | --- |
| GWAS  CMA | 1.106 | 0.996 | 1.030 |
| eGFRcr_eGFRcys_sRAGE |  |  | 1.094 |
| eGFRcr_eGFRcys |  | 1.095 |  |
| eGFRcr_sRAGE |  |  | 1.094 |
| eGFRcys_sRAGE |  |  | 1.094 |

*Note*: eGFR = estimated glomerular filtration rate (eGFR), eGFRcr = eGFR from serum creatinine, eGFRcys = eGFR from serum cystatin C, sRAGE = serum levels of soluble receptor for advanced glycation end products.

**Supplemental Table 4.** Tetrachoric correlations of CMA for kidney function traits and sRAGE

| Traits | eGFRcr | eGFRcys | eGFRcr, eGFRcys |
| --- | --- | --- | --- |
| eGFRcys | 0.012 |  |  |
| sRAGE | 0.012 | 0.016 | 0.016 |

*Note*: eGFR = estimated glomerular filtration rate (eGFR), eGFRcr = eGFR from serum creatinine, eGFRcys = eGFR from serum cystatin C, sRAGE = serum levels of soluble receptor for advanced glycation end products.

**Supplemental Table 14.** Comparison of CKD between LLFS and random US populations

|  | LLFS | CDC | LLFS | | CDC |
| --- | --- | --- | --- | --- | --- |
| Mean age (years)  Age range | 54.5  [50-59] | 54.5 [45-64] | 64.5  [60-69] | 74.5  [70-79] | 65+ |
| CKDcr (%) | 4.4 | 12.3 | 11.7 | 32.3 | 33.7 |

*Note*: The LLFS values are from Supplemental Table 2. The CDC reference is “Centers for Disease Control and Prevention. Chronic Kidney Disease in the United States, (2023). Atlanta, GA: US Department of Health and Human Services, Centers for Disease Control and Prevention; 2023 (<https://www.cdc.gov/kidney-disease/media/pdfs/CKD-Factsheet-H.pdf> )”.

**Supplemental Figure 1.** Distribution of creatinine, cystatin C, and sRAGE by 10-year age group

boxplots showing interquartile ranges and medians shown in **Supplemental Table 2**


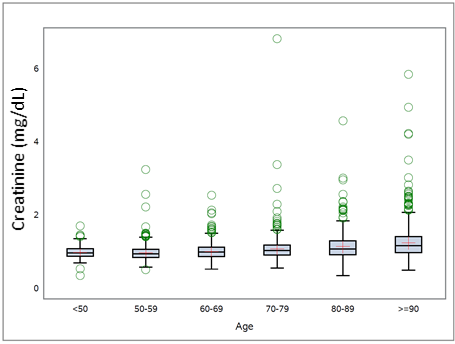
Serum Creatinine


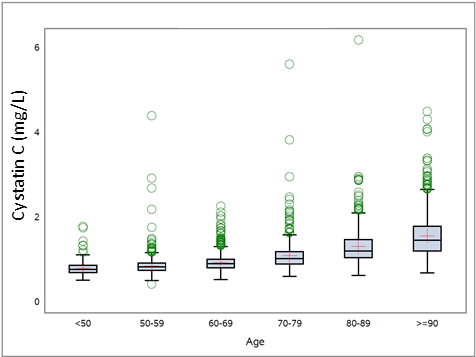
Serum Cystatin C


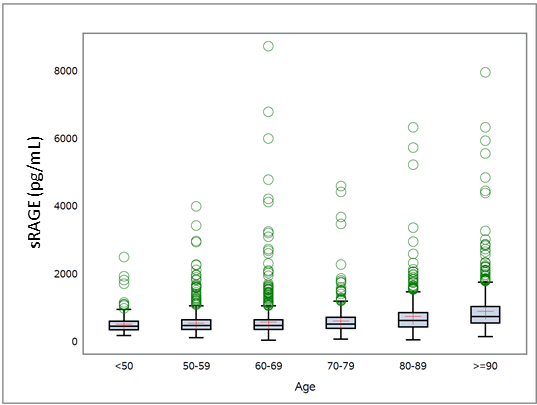
Serum sRAGE

**Supplemental Figure 2.** GWAS quantile-quantile plots of observed versus expected -log_10_ (*p*-value) of kidney function (eGFRcr and eGFRcys) and sRAGE

eGFRcr


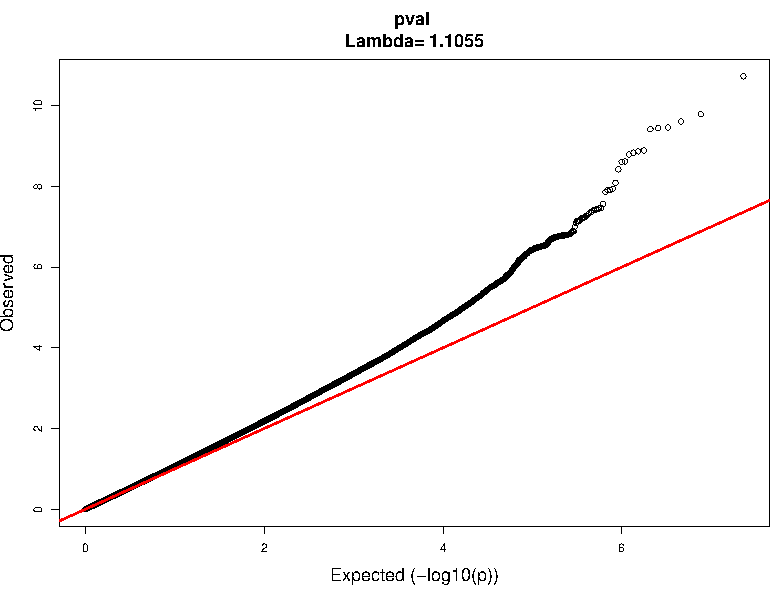


0 2 4 6 8 10

Observed -log_10_ (*p*-value)

Observed -log_10_ (*P*-value)

Expected -log_10_ (*p*-value)

0 2 4 6


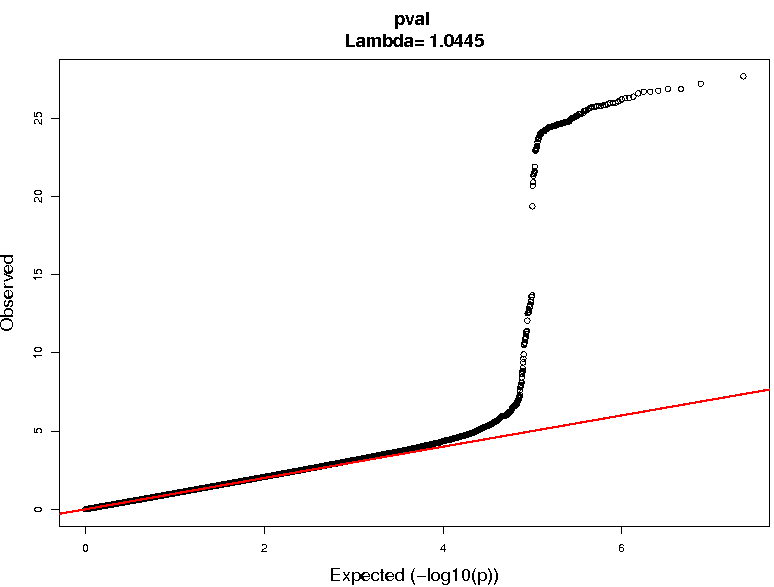


Observed -log_10_ (*p*-value)

0 5 10 15 20 25

Expected -log_10_ (*p*-value)

0 2 4 6

eGFRcys


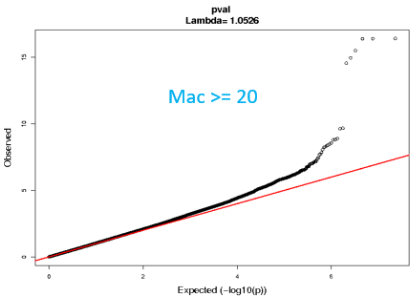


sRAGE

Expected -log_10_ (*p*-value)

Observed -log_10_ (*p*-value)

0 5 10 15

0 2 4 6


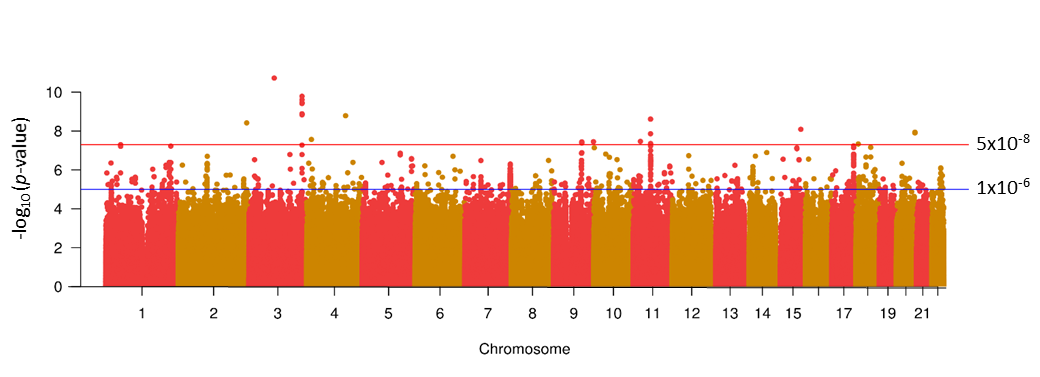
**Supplemental Figure 3.** Manhattan plots of GWAS for kidney function traits and sRAGE

eGFRcr


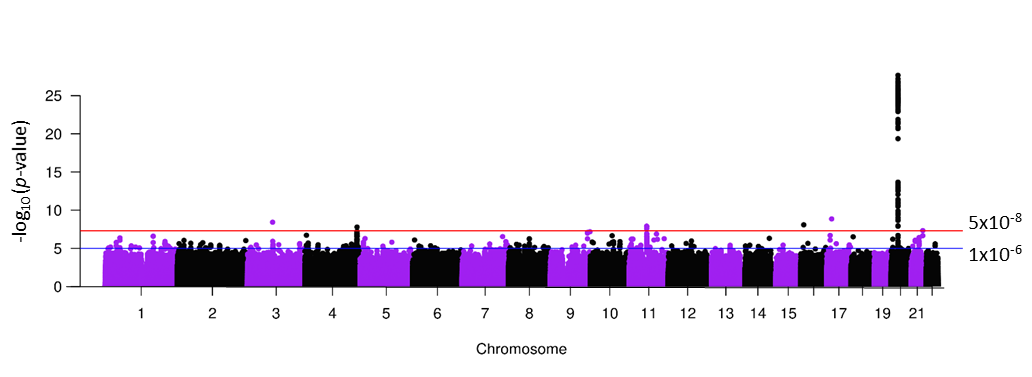


eGFRcys


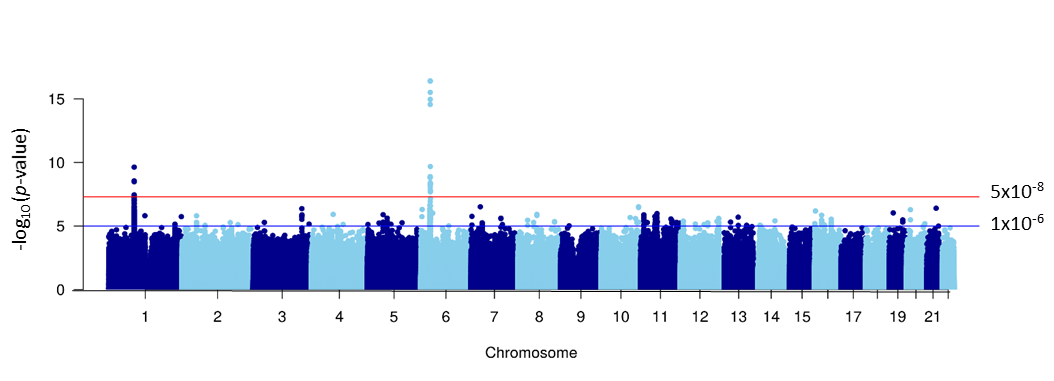


sRAGE

**Supplemental Figure 4.** Manhattan plots of CMA GWAS for kidney function traits and sRAGE


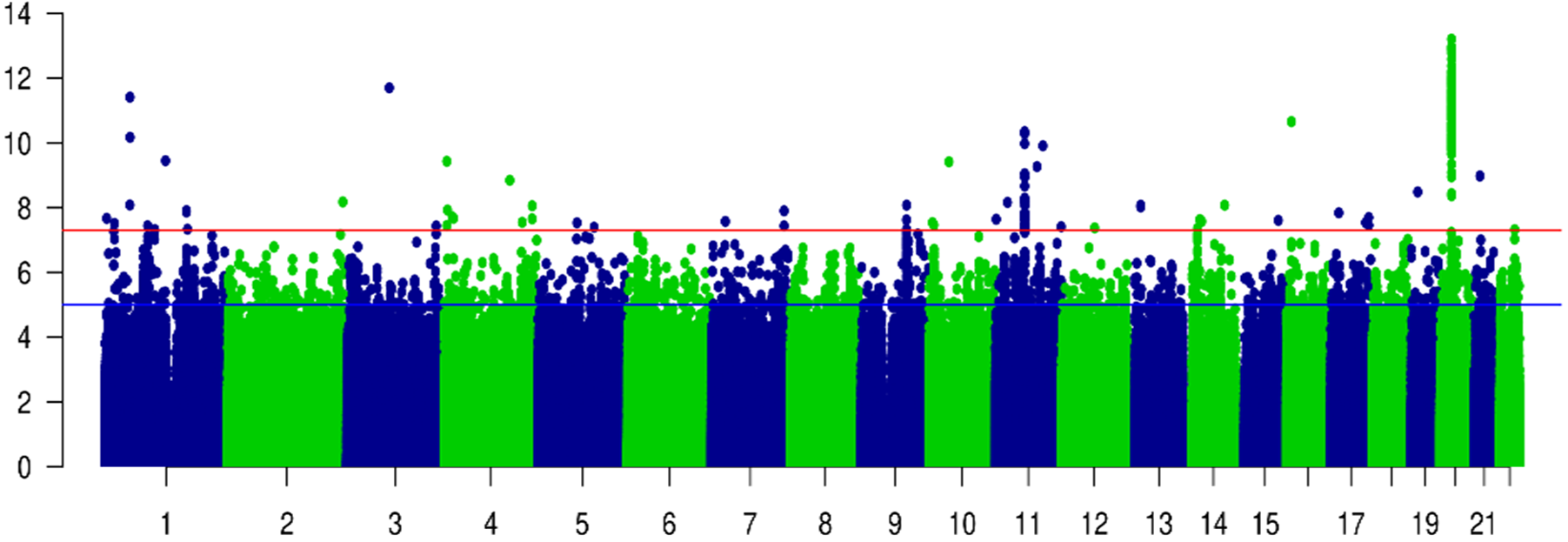


CMA of eGFRcr, eGFRcys, and sRAGE

Chromosome

-log_10_ (*p*-value)

5x10^-8^

1x10^-6^


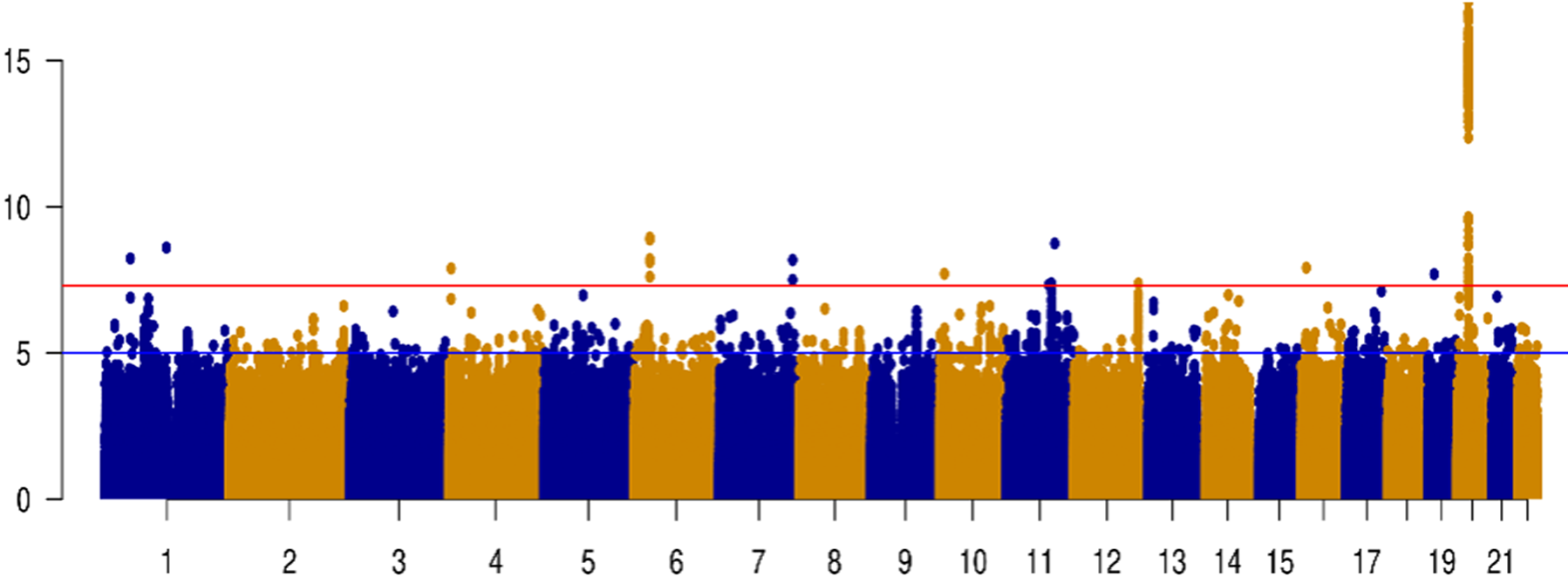


CMA of eGFRcys and sRAGE

Chromosome

-log_10_ (*p*-value)

5x10^-8^

1x10^-6^


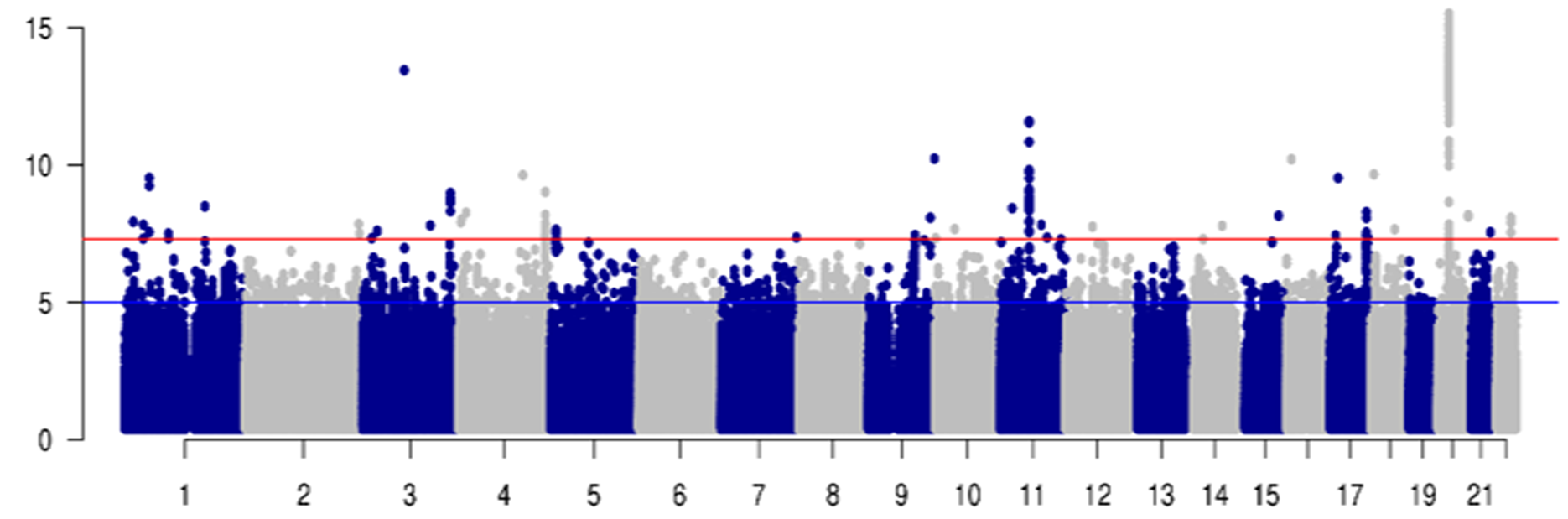


CMA of eGFRcr and eGFRcys

Chromosome

-log_10_ (*p*-value)

5x10^-8^

1x10^-6^


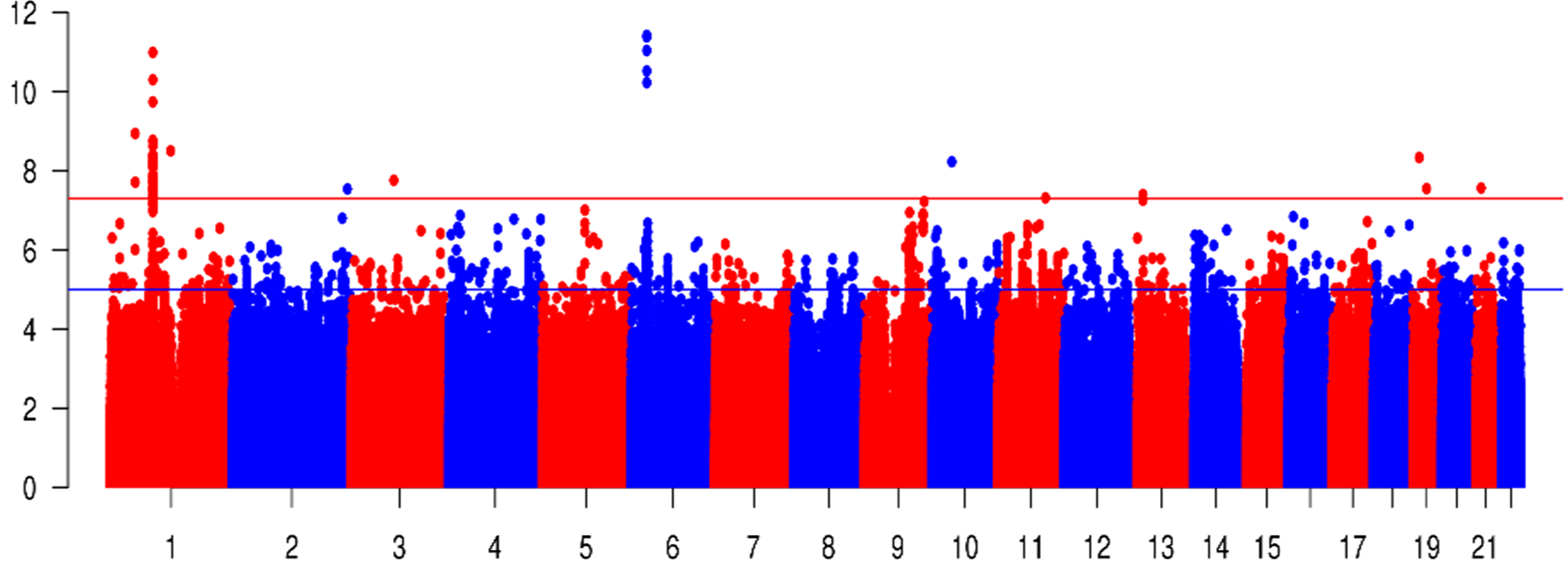


CMA of eGFRcr and sRAGE

Chromosome

-log_10_ (*p*-value)

5x10^-8^

1x10^-6^

**Supplemental Figure 5.** Locuszoom plots of novel CMA GWAS


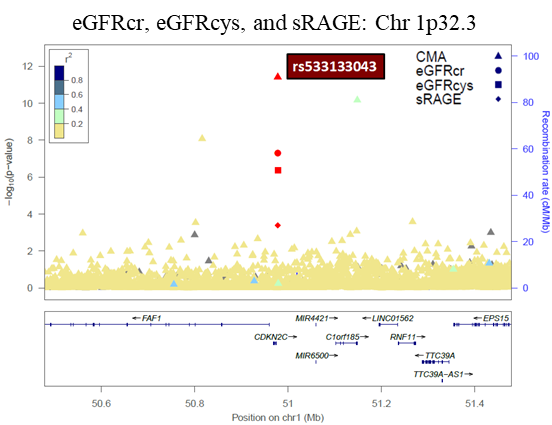

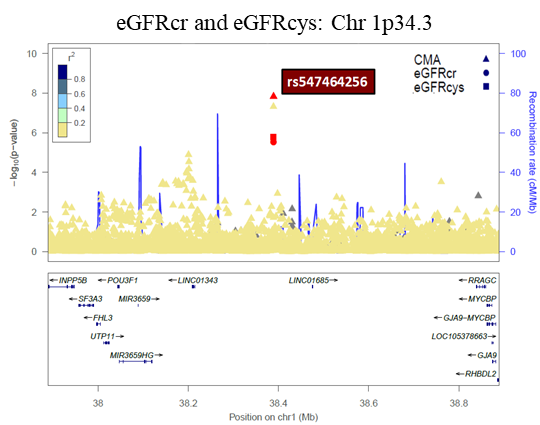


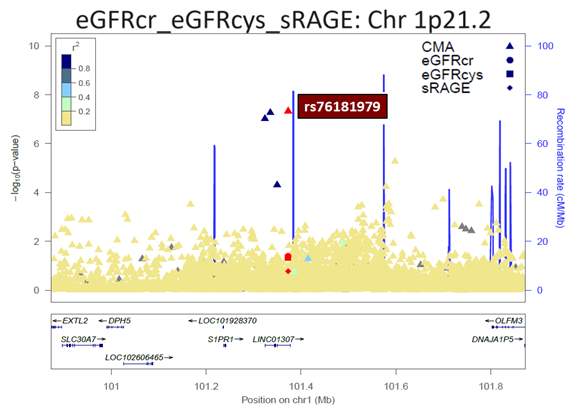

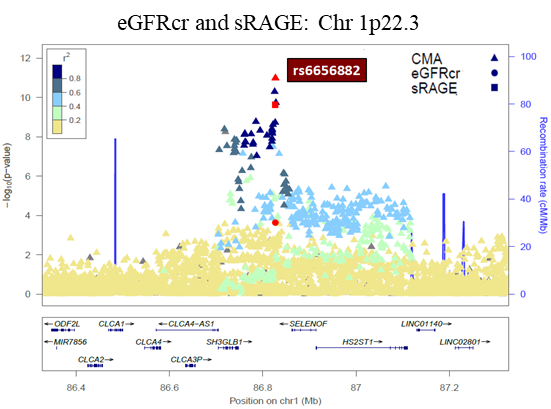

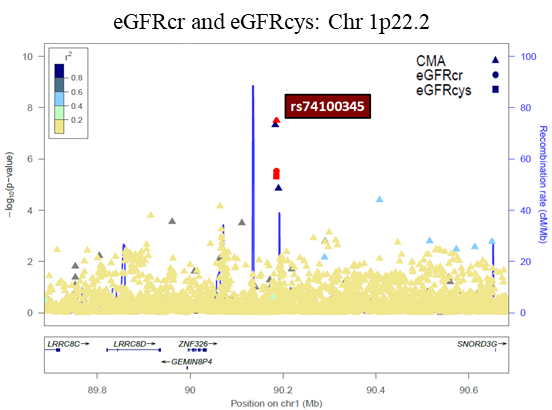

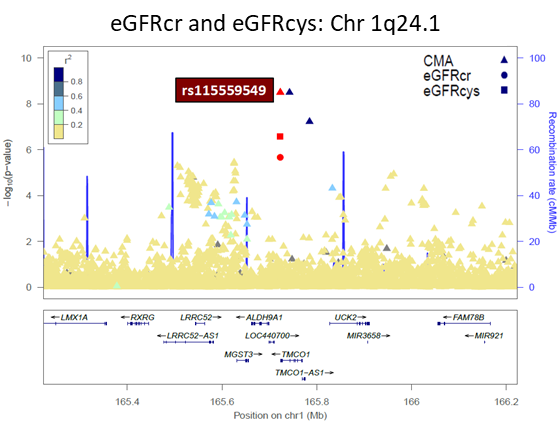


**
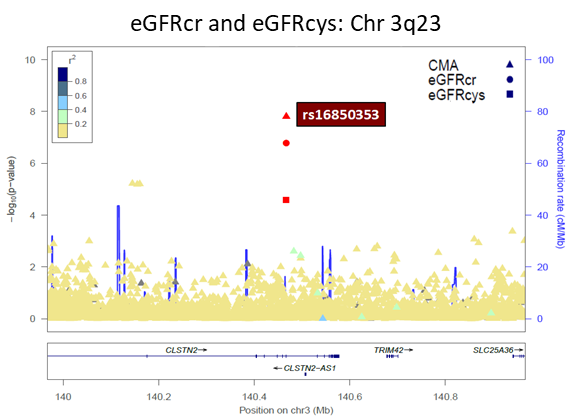

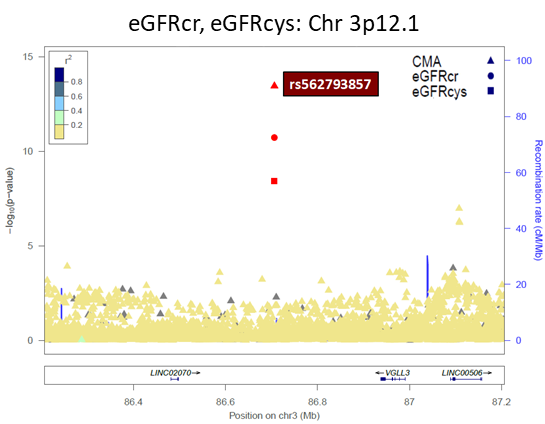

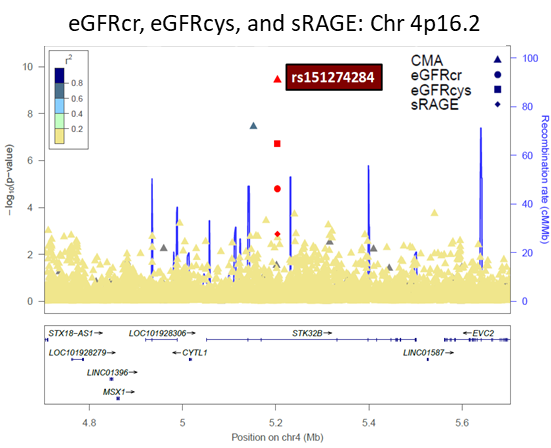

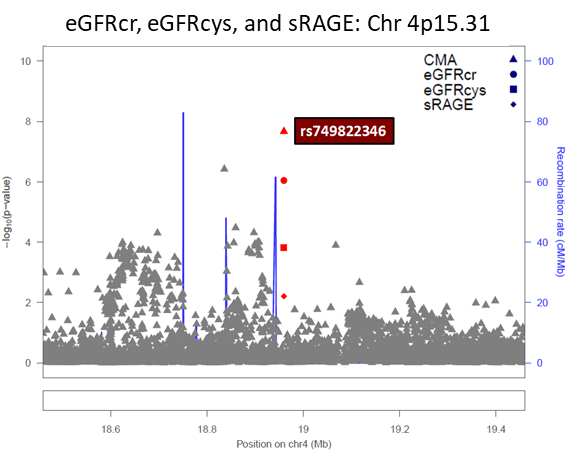

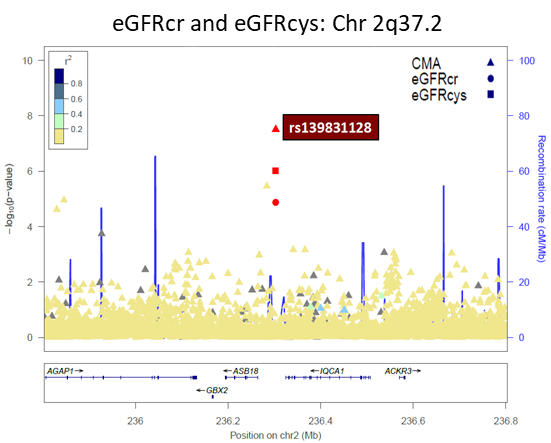

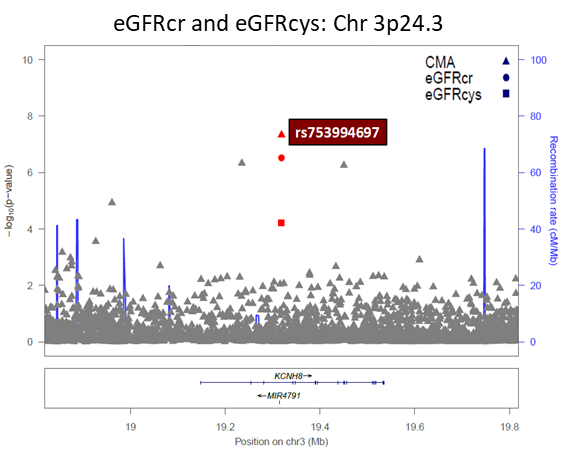
**

**
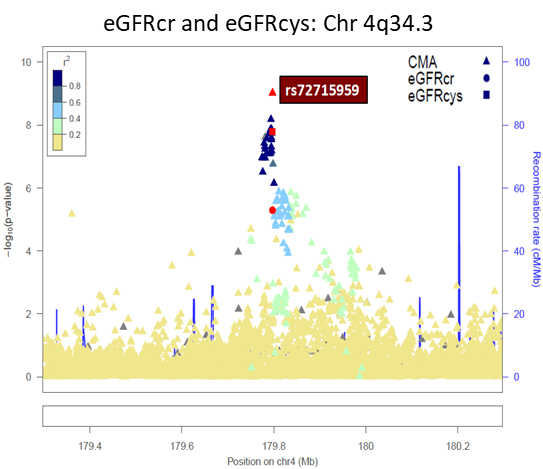

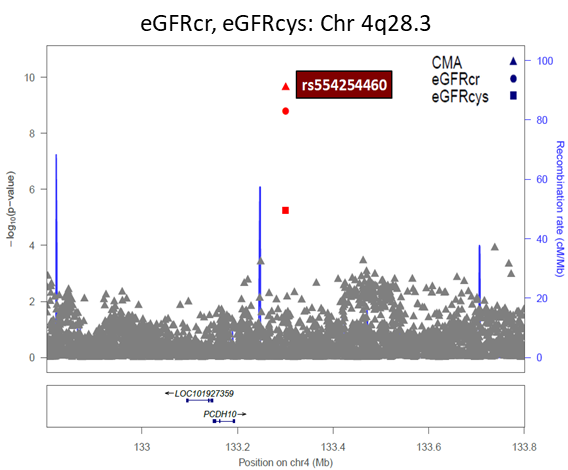
**

**
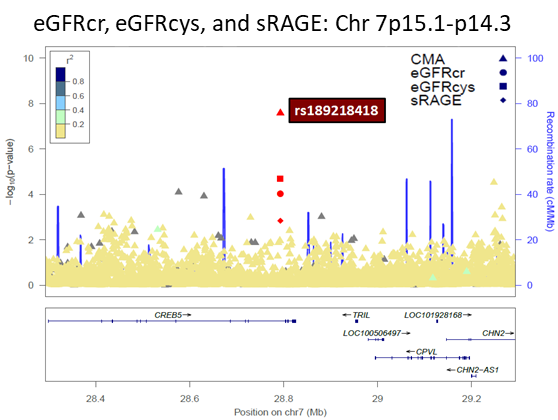

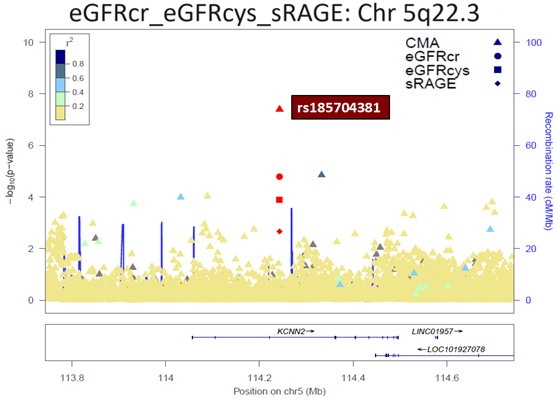

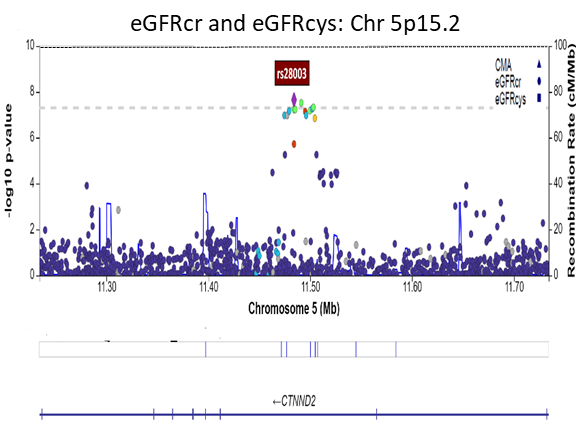

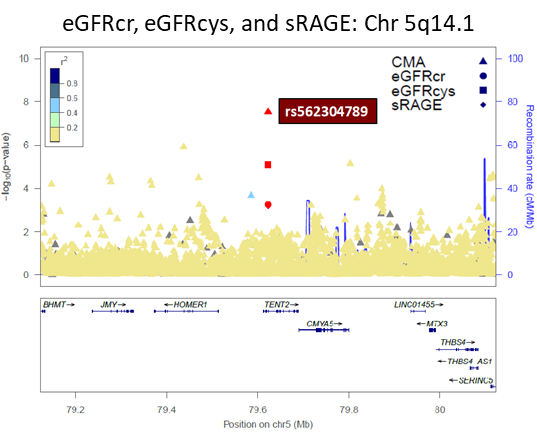
**

**
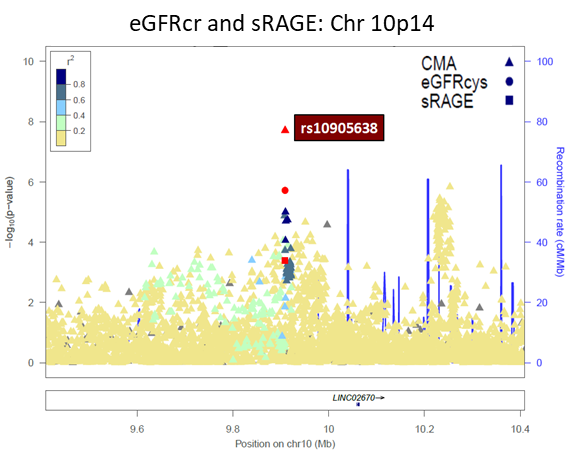

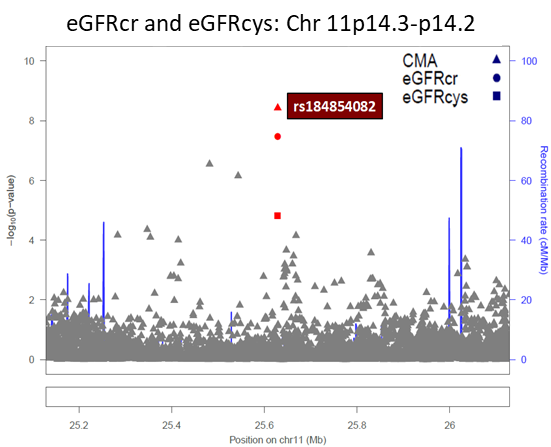

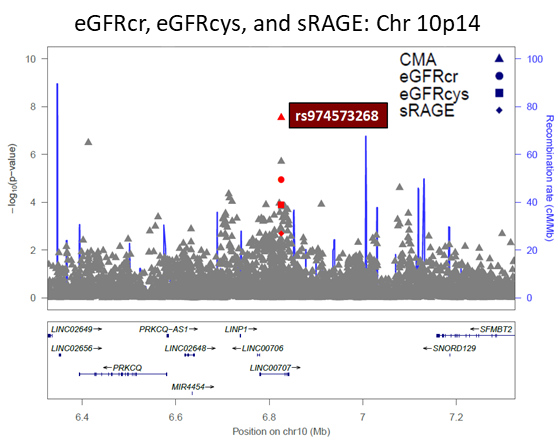

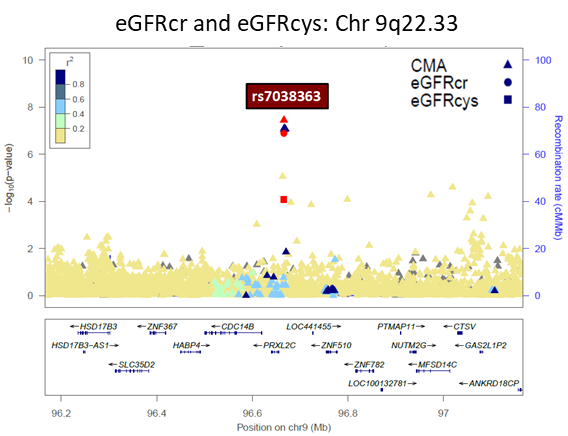

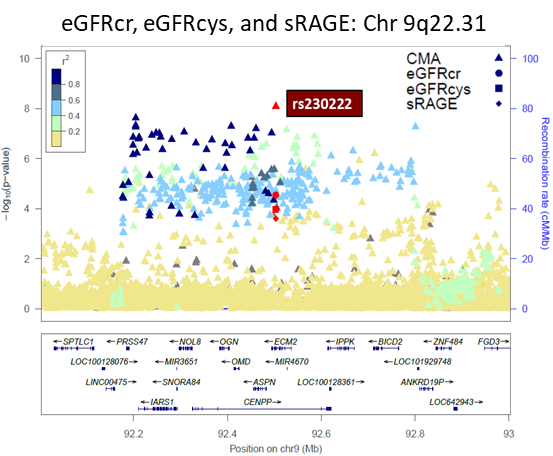

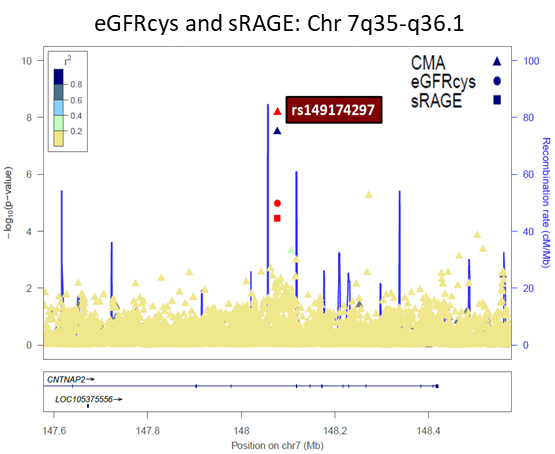
**

**
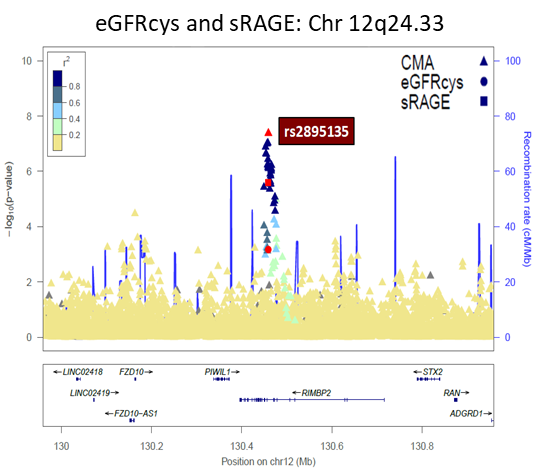

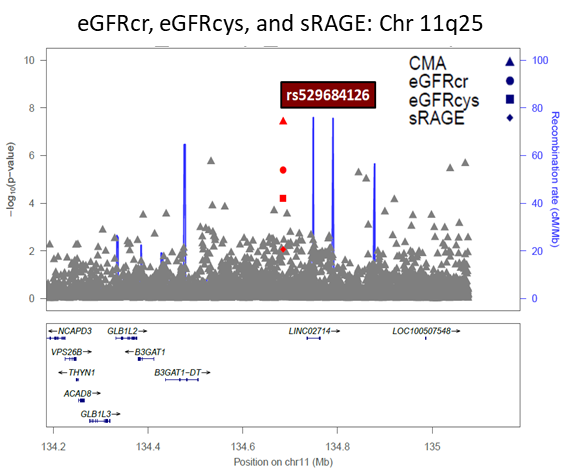

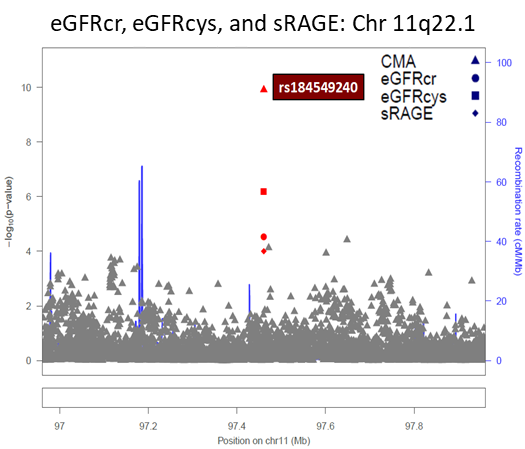

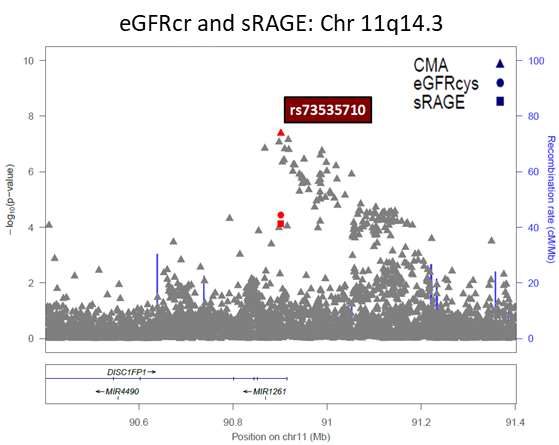

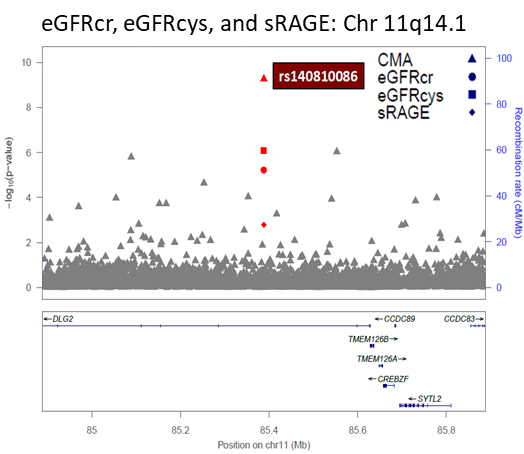

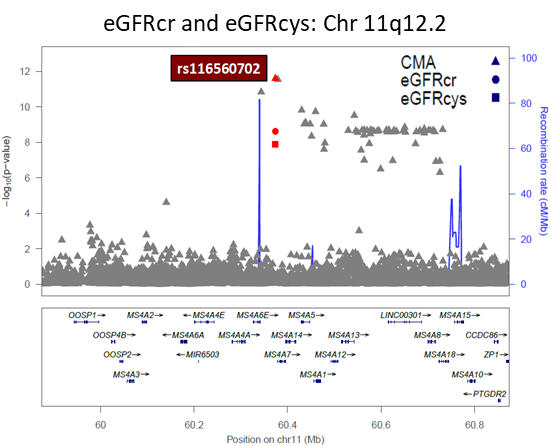
**

**
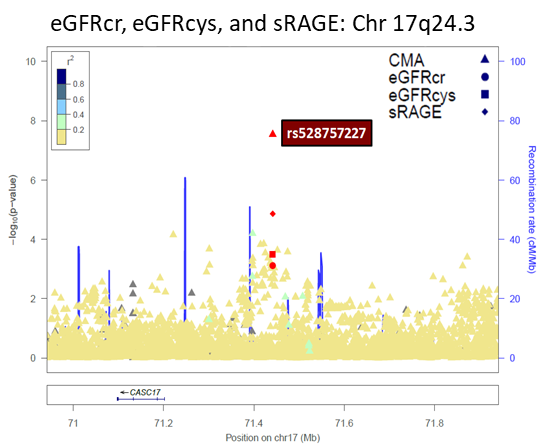

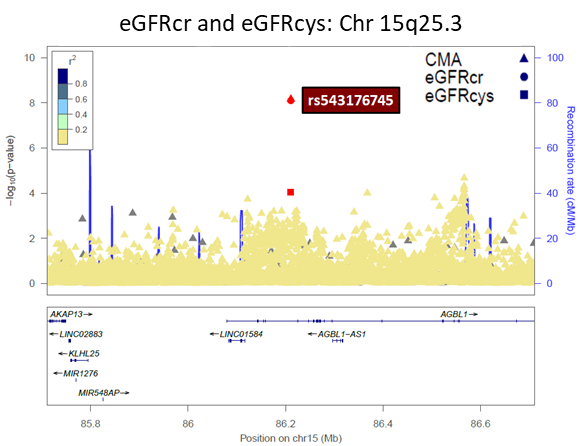

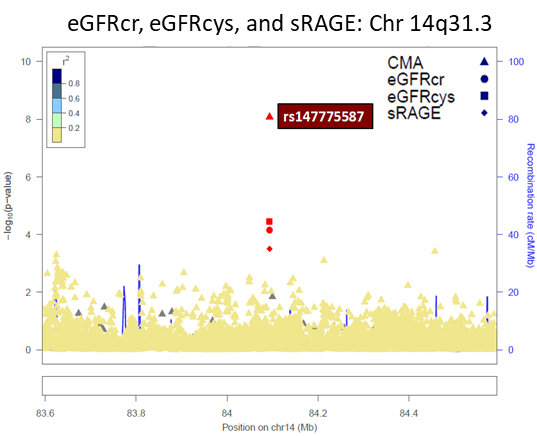

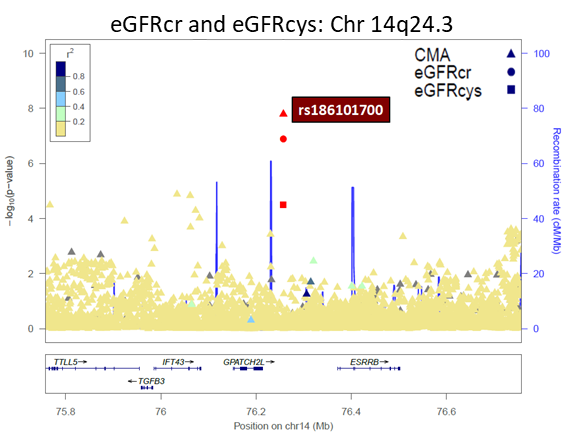

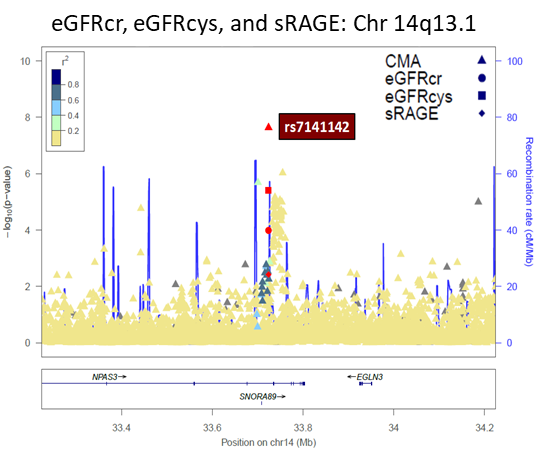

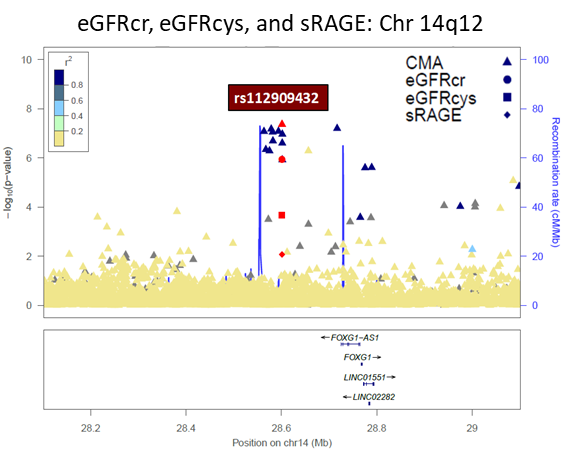
**

**
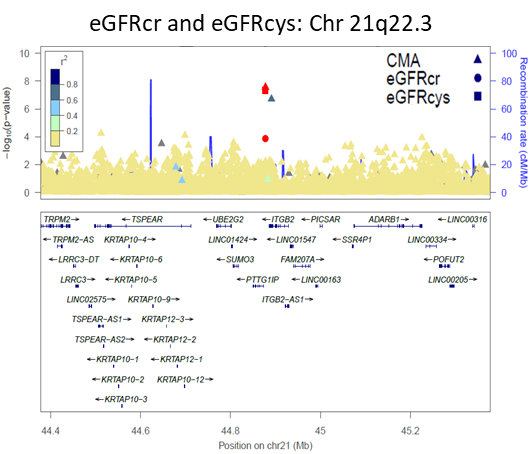

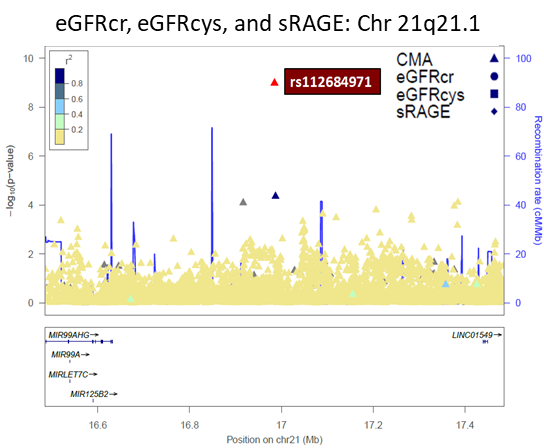

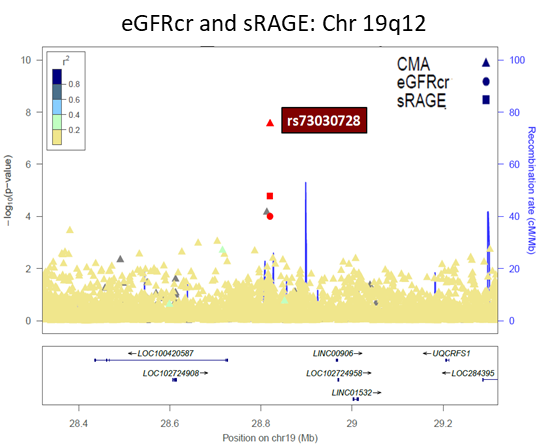

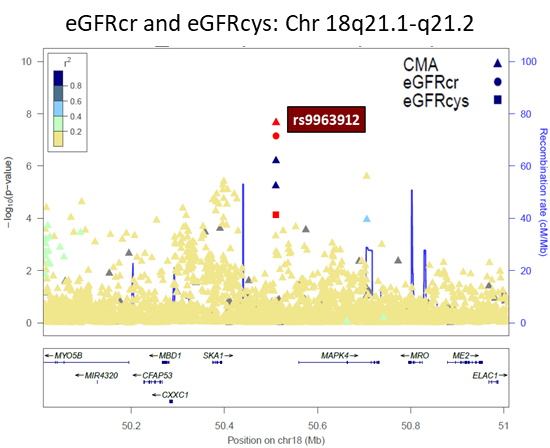

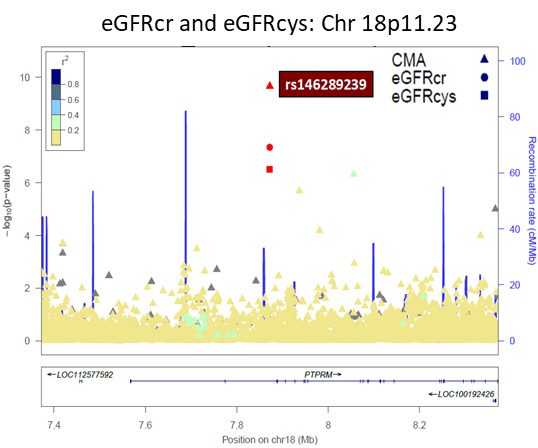

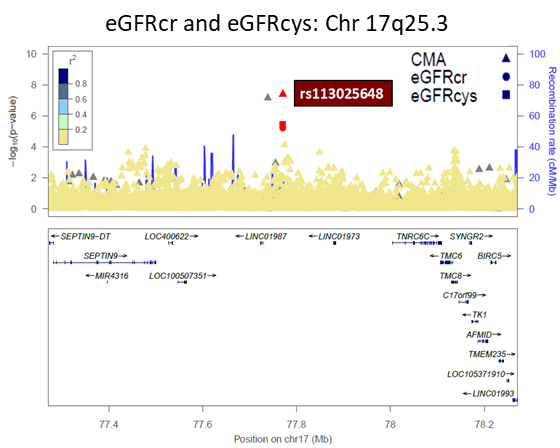
**


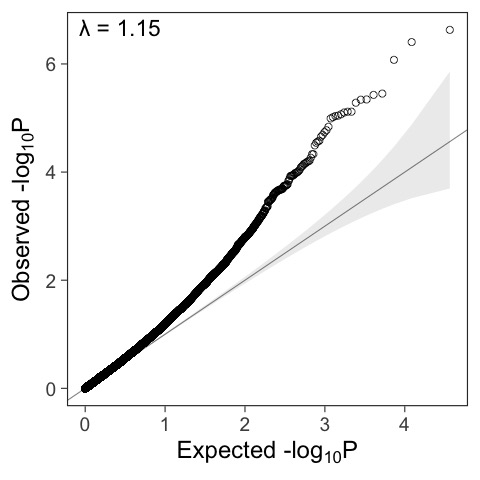

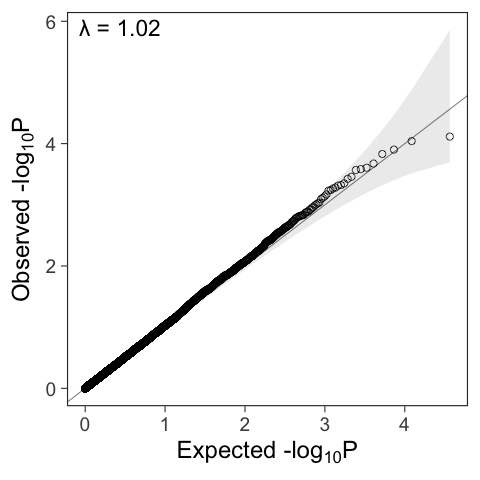

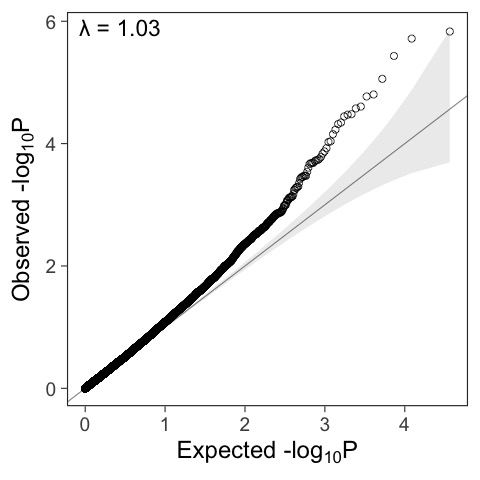
**Supplemental Figure 6.** TWAS quantile-quantile plots of observed versus expected -log_10_ (*p*-values)

eGFRcys

eGFRcr

sRAGE

**Supplemental Figure 7.** Manhattan plots of TWAS for kidney function traits and sRAGE

eGFRcr

**
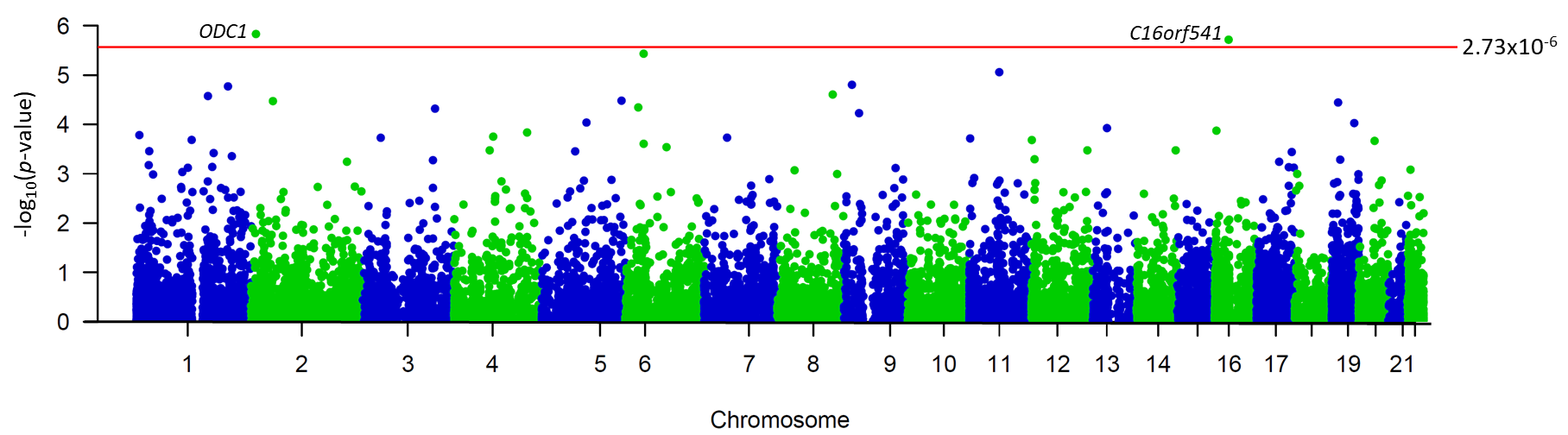
**

eGFRsys

**
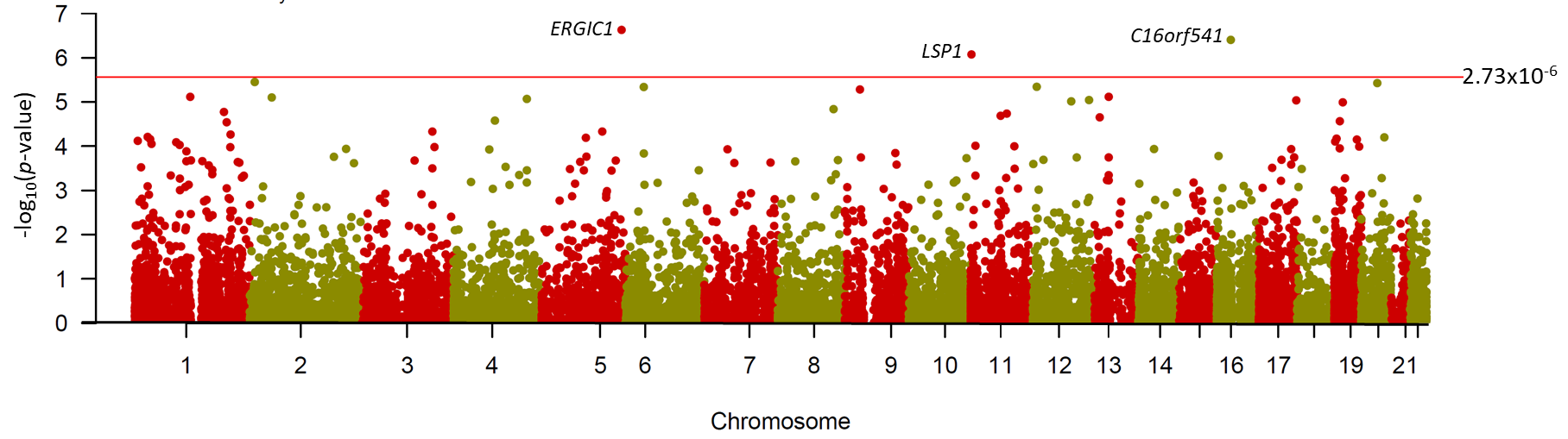
**

sRAGE

**
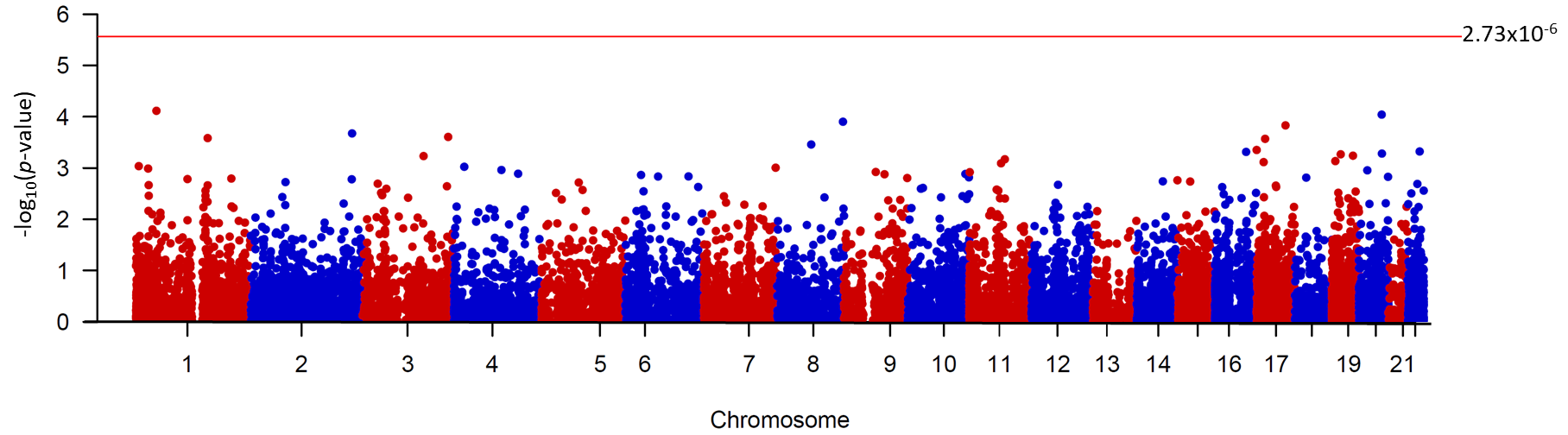
**
